# Antielastase and Antihyaluronidase Activity of Isomangiferin, a Xanthone with Strong Antioxidant Properties

**DOI:** 10.3390/ijms27146258

**Published:** 2026-07-14

**Authors:** Anna Hering, Krzysztof Szafrański, Barbara Mikolaszek, Karolina Milewska, Myroslava Kucher, Aleksandra Komala, Aleksandra Kowalewska, Justyna Stefanowicz-Hajduk

**Affiliations:** 1 Department of Biology and Pharmaceutical Botany, Medical University of Gdansk, 80-416 Gdansk, Poland; justyna.stefanowicz-hajduk@gumed.edu.pl; 2 Department of Organic Chemistry, Medical University of Gdansk, 80-416 Gdansk, Poland; krzysztof.szafranski@gumed.edu.pl; 3 Department of Pharmaceutical Technology, Medical University of Gdansk, 80-416 Gdansk, Poland; barbara.mikolaszek@gumed.edu.pl; 4 Department of Built Environment and Energy Technology, School of Engineering, Linnæus University, SE-351 95 Växjö, Sweden; 5 Faculty of Pharmacy, Medical University of Gdansk, 80-416 Gdansk, Poland; 4529192@gmail.com (M.K.); ola.komala2000@gmail.com (A.K.); a.kowalewska315@gmail.com (A.K.)

**Keywords:** elastin fiber, mangiferin, antiaging activity, free radicals, ECM enzymes, keratinocytes

## Abstract

Isomangiferin is a natural C-glycosylated xanthone, an isomer of mangiferin. The biological properties of isomangiferin are not clearly known. The antioxidant effect of this xanthone was assessed with 2,2′-azino-bis(3-ethylbenzothiazoline-6-sulfonic acid) (ABTS), 2,2-diphenyl-1-picrylhydrazyl (DPPH), and ferric-reducing antioxidant power (FRAP) tests. Isomangiferin’s cytoprotective effects against menadione in human keratinocytes HaCaT were evaluated via an MTT (3-(4,5-dimethylthiazol-2-yl)-2,5-diphenyltetrazolium bromide) test. Additionally, the inhibition of elastase and hyaluronidase by isomangiferin was tested spectrophotometrically. Antielastase activity was analyzed in silico and by evaluating the degradation of elastin fibers and elastin in tendons using image analysis. The results indicated that the antioxidant properties of the tested xanthone were stronger than those of ascorbic acid (the IC_50_ values for isomangiferin and ascorbic acid were 27.04 ± 1.8 and 164.6 ± 3.29 µM; 9.57 ± 0.05 and 35.83 ± 0.45 µM; and 70.44 ± 1.4 and 315.85 ± 4.49 µM in ABTS, DPPH and FRAP tests, respectively). Isomangiferin at a concentration of 95 µM (40 µg/mL) effectively protected HaCaT cells from death. This compound also demonstrated a moderate ability to inhibit hyaluronidase activity. The ability to inhibit elastase was demonstrated via image analysis and supported by docking studies, which indicated that isomangiferin binds with elastase not directly within the catalytic site but in the S4 subpocket. This study shows a promising direction for the development of new agents that mitigate oxidative stress-induced aging by protecting both elastin fibers and hyaluronic acid from degradation.

## 1. Introduction

**Figure 1:**
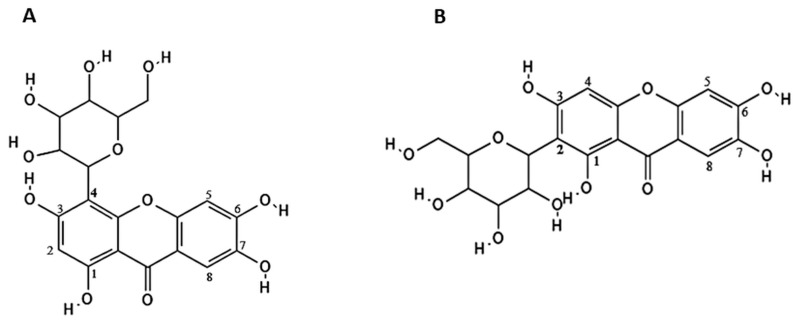
(**A**) Isomangiferin (1,3,6,7-tetrahydroxyxanthone-C4-β-D-glucoside) and (**B**) mangiferin (1,3,6,7-tetrahydroxyxanthone-C2-β-D-glucoside).

Isomangiferin (Figure 1A) is an isomer of mangiferin (Figure 1B) that differs in the location of glucose on the xanthone skeleton. These biomolecules are commonly found together, although isomangiferin is present at significantly lower amounts. The main source of mangiferin and its isomers, like isomangiferin and homomangiferin, is mango (*Mangifera indica* L., Anacardiaceae), where the xanthones are sourced mainly from leaves [1,2]. These compounds can also be found in plants of *Cyclopia* sp. (Fabaceae), more commonly known as honeybush. The leaves and stems of this polyphenol-rich endemic plant, found only in Africa in the Cape Provinces, are used by local populations to make a health-promoting herbal tea consumed to treat many ailments [3,4]. Mangiferin, which is the main component of extracts obtained from both mango and honeybush, has many biological properties, mostly due to its strong antioxidant properties. It has been proven that the molecule shows radioprotective, cardiotonic, antidiabetic, neuroprotective, and hepatoprotective properties and can inhibit extracellular matrix (ECM) enzymes, among other functions [5,6,7,8]. Mangiferin was tested using the spectroscopic method to determine its antielastase activity. The results revealed that elastase activity was inhibited by mangiferin in a dose-dependent manner. The IC_50_ of mangiferin was 139.64 ± 9.34 μM, while an increase in concentration up to 473 μM caused the complete inhibition of elastase [9].

According to the literature data, only a few studies have been conducted on isomangiferin. Since the plant extracts that contain mangiferin also contain isomangiferin and homomangiferin, researchers determined that the quantity of those xanthones and biological activity were mainly attributed to mangiferin. However, each of these compounds may have different biological properties. This may be due to the fact that the C-glucoside substituent is located in a different C-location in the compound structure. Thus, the molecule may interact with proteins and other biological macromolecules in a different way. For example, one conformation may bind to the enzyme’s active center, while others only bind to the surface [10].

So far, it has been indicated that isomangiferin can be effective in treating breast cancer [11]. Another study has indicated the utility of isomangiferin in diabetes treatment by lowering blood sugar and insulin resistance. Isomangiferin can also be used in complications of diabetes like diabetic nephropathy, including kidney inflammation [12]. In bone differentiation research, isomangiferin promoted BMSC motility and influenced the AMP-activated protein kinase/acetyl-CoA carboxylase (AMPK/ACC) pathway [13]. There are no studies on the action of isomangiferin on ECM enzymes, including elastase and hyaluronidase.

The presence of elastin determines the tissue’s ability to stretch and maintain elasticity. Elastin fibers can be linearly stretched more than twice their original size, and upon release of the tension, they return to their original dimensions. Elastin is most abundant in the skin, lungs, and blood vessels. The structure and quantity of elastin in different types of tissues depend on their location and local hemodynamic conditions. The development of microscopic techniques has revealed complex networks of fine elastin fibers in different tissues and body parts, such as small blood vessels, cartilage, intervertebral discs, and even adipose tissue and tendons [14,15,16]. Elastin is involved in the development and progression of many cardiovascular and pulmonary diseases through changes in its expression or structural degradation [17]. Elastase, an enzyme responsible for elastin degradation, is released during inflammation, degrading connective tissue proteins like elastin and collagen, which leads to tissue damage and acceleration of inflammation processes [14,17,18,19].

Hyaluronic acid is a glycosaminoglycan, an important component of the extracellular matrix and a popular filler for aesthetic procedures. It is composed of repeating units of D-glucuronic acid and N-acetylglucosamine, which are linked by sugar bonds. Hyaluronidase is an endoglycosidase that degrades hyaluronic acid into smaller molecules and increases tissue permeability. These degraded fragments can act as signals, triggering further inflammatory processes [20,21,22,23].

Inflammation can also be caused by the action of free radicals that have a destructive effect on the body. Their unpaired electrons can cause degradation of macromolecules, including DNA, enzymes, and proteins, and change their function. An additional target for free radicals is lipid membranes, which undergo peroxidation and decomposition [24]. Natural antioxidant mechanisms may be insufficient when exposed to external free radicals derived from food, smog, or UV radiation. The use of plant raw materials or individual metabolites with antioxidant properties effectively supports the body’s defense mechanisms in the fight against free radicals; therefore, the search for compounds with such properties is extremely important.

This work aims to evaluate the antioxidant properties of isomangiferin and its preliminary inhibitory effect on ECM enzymes, elastase and hyaluronidase, representing the first study on this compound as a potential antielastase and antihyaluronidase agent.

## 2. Results

### 2.1. Antioxidant Activity

ABTS and DPPH radicals were used to check the antioxidant activity of isomangiferin compared to standard ascorbic acid. In order to assess the reducing power of isomangiferin, the FRAP test was utilized, which is a rapid assay that provides reproducible results for direct determination of the reducing properties of a pure compound.

In all three tests, isomangiferin represented higher free radical scavenging abilities and reducing properties than standard ascorbic acid (Table 1). The ABTS results showed the biggest differences, with IC_50_ values = 27.04 ± 1.8 and 164.6 ± 3.29 µM (IC_50_ 11.42 ± 0.76 and 28.99 ± 0.58 µg/mL) for isomangiferin and ascorbic acid, respectively. In the DPPH test, the IC_50_ value of isomangiferin was 9.57 ± 0.05 µM—3.8-fold lower than the IC_50_ value of ascorbic acid, which was 35.83 ± 0.45 µM (4.04 ± 0.02 and 6.31 ± 0.08 µg/mL, respectively).

In the FRAP assay, isomangiferin exhibited almost 4.5-fold greater antioxidant activity than the standard substance. The IC_50_ values were 70.44 ± 1.4 and 315.85 ± 4.49 µM (29.75 ± 0.59 and 55.63 ± 0.79 µg/mL), respectively.

### 2.2. Protective Activity on HaCaT Cells

**Figure 2:**
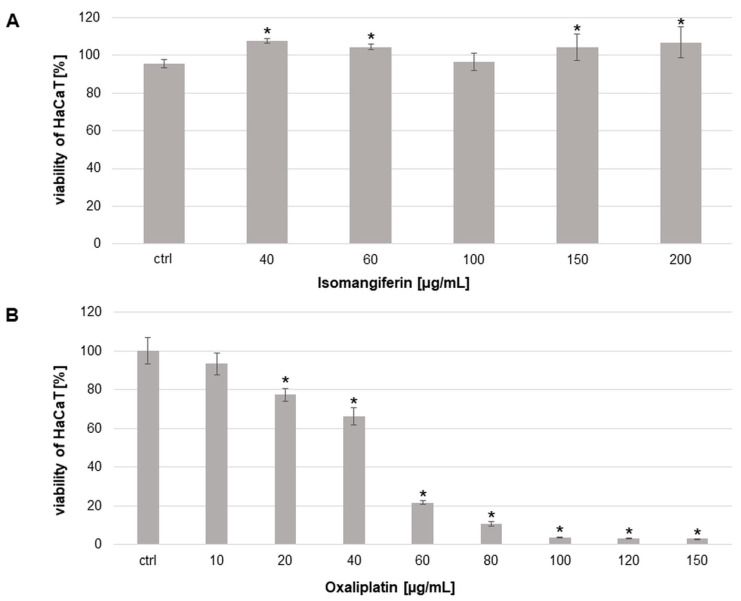
The effect of isomangiferin (**A**) and oxaliplatin (**B**) on HaCaT viability. Oxaliplatin was used as a positive control. The results are presented as the mean values with standard deviations (±SDs) calculated from three independent experiments in three repetitions (*n* = 9). Error bars indicate standard deviations. Significant differences relative to the control are marked with an asterisk “*” (Welch’s *t*-test, *p* < 0.05).

The protective activity of isomangiferin was confirmed via an in vitro test using the HaCaT cell line. The cells were incubated with the compound for 24 h, and their viability was assessed with the MTT assay (Figure 2A).

It was observed that, within the tested concentration range (40–200 µg/mL), isomangiferin did not exhibit a cytotoxic effect against keratinocytes. Cell survival in all isomangiferin treatments remained high, similar to the control cells. Oxaliplatin, a well-known cytostatic, served as a positive control, exhibiting an IC_50_ = 45.75 ± 1.95 µg/mL (Figure 2B).

**Figure 3:**
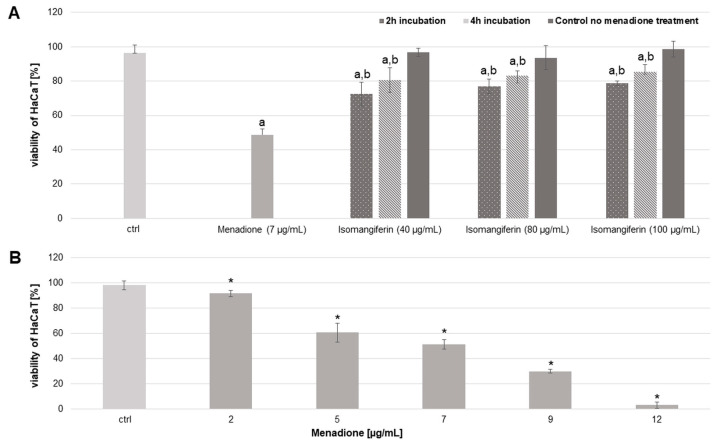
The viability of HaCaT cells in the presence of menadione and/or isomangiferin, obtained via the MTT test. (**A**) In the first step, the cells were incubated with isomangiferin (40, 80, and 100 µg/mL) for 2, 4, or 24 h. The control was the untreated cells. After 2 and 4 h of incubation, menadione was added to the cells with isomangiferin for 24 h (gray dots and gray lines columns). (**B**) The viability of HaCaT cells in the presence of menadione. The results are presented as the mean values with standard deviations (±SDs) calculated from three independent experiments across three repetitions (*n* = 9). Error bars indicate standard deviations. Significant differences relative to the control are marked with letters “^a^” (in comparison to the control) and “^b^” (in comparison to menadione 7 µg/mL) (Welch’s *t*-test, *p* < 0.05). Significant differences relative to the control are marked with an asterisk “*” (Welch’s *t*-test, *p* < 0.05).

In order to determine isomangiferin’s protective effect on cell survival, menadione was used (Figure 3A). This substance, at a concentration of 7 µg/mL, caused a significant reduction in the HaCaT viability (up to 50%, Figure 3B). The analysis involved incubating keratinocytes for 2 or 4 h with three different concentrations of isomangiferin, following menadione treatment. The assay showed that isomangiferin itself (40, 80, 100 µg/mL) did not affect cell viability. In turn, isomangiferin protected the cells from menadione-induced death, and this effect was dependent on both concentration and incubation time. The lowest concentration of isomangiferin showed the lowest protective effect, with cell viability results of 72.32 ± 6.95 and 80.43 ± 7.47% after 2 and 4 h incubation, respectively. At a higher concentration (80 µg/mL), the results were 76.8 ± 4.21 and 83.01 ± 2.75% after 2 and 4 h, respectively. The highest protective effect was observed when an isomangiferin concentration of 100 µg/mL was used, with viability results of 78.81 ± 1.32 and 85.38 ± 4.14% after 2 and 4 h, respectively. The obtained data also showed that time has a clear influence on the survival effect of isomangiferin. The longer the exposure to isomangiferin, the stronger the effect against menadione in cells at each of the analyzed isomangiferin concentrations. In order to confirm the hypothetical antioxidant effect of isomangiferin on living cells, further detailed studies are necessary.

### 2.3. Antielastase Activity

Destruction of elastin promotes the development and progression of various pathological conditions, including obstructive pulmonary disease, emphysema, atherosclerosis, metabolic syndrome, and cancer [25]. Thus, inhibition of this enzyme plays an important role in limiting inflammation cascades.

#### 2.3.1. Inhibition Assay Results

**Figure 4:**
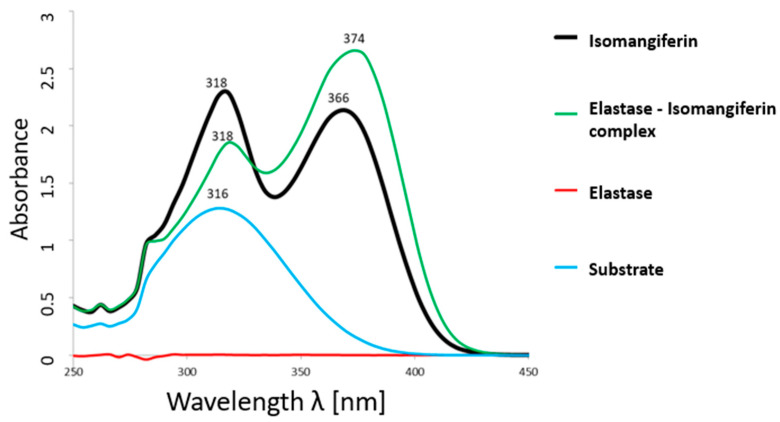
UV maxima of main components of reaction mixture after background subtraction (buffer spectrum). Red line: elastase (25 µg/mL); blue line: substrate (SANA, 1 mg/mL), λ_max_—316 nm; black line: isomangiferin (100 µg/mL), λ_max_—318 nm and λ_max_—366 nm; green line: elastase (25 µg/mL): isomangiferin (100 µg/mL) complex, λ_max_—318 nm and 374 nm.

Isomangiferin was tested spectrophotometrically to establish its inhibitory potential against elastase. The method described by Thring et al. [26] proved to be effective in studying mangiferin’s inhibition of elastase activity [9]. In the case of isomangiferin, monitoring product formation in its presence precluded the interpretation of absorbance changes. This may be due to the formation of a complex between the enzyme and isomangiferin. As a consequence, measuring the IC_50_ using a spectrophotometric method becomes impossible. Therefore, the UV spectra (from 250 to 600 nm) of the main components of the reaction mixtures were analyzed. Figure 4 proves that the standard spectroscopic procedure is ineffective due to overlapping absorbance maxima. In this assay, constant monitoring of the increase in absorption at 410 nm was carried out, which corresponds to product formation (SANA degradation). The substrate (SANA) had one peak at λ_max_ = 316 nm, and isomangiferin had two peaks with the maxima at 318 nm and 366 nm. After incubation of the elastase with isomangiferin, two peaks were observed at 318 nm and 374 nm. The first maximum can be related to isomangiferin, although a decrease in absorption was observed. The second isomangiferin peak (366 nm) in the isomangiferin–elastase mixture increased significantly and shifted to 374 nm, indicating the formation of a complex between the enzyme and isomangiferin. Due to the overlapping absorbance maxima of both the substrate (316 nm) and the product (410 nm) with the complex between the enzyme and the isomangiferin (318 nm and 374 nm), interpretation of the results of the enzymatic reaction was impossible.

**Figure 5:**
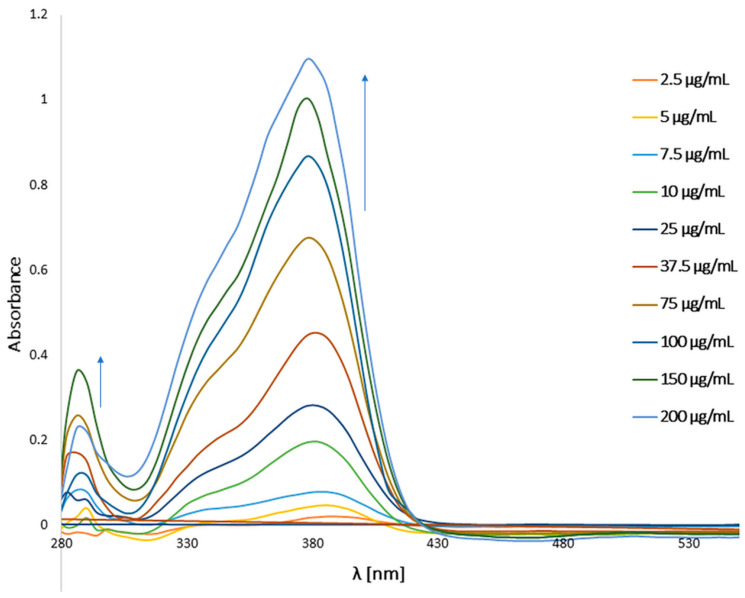
Complex formation of isomangiferin (100 µg/mL) with elastase (2.5−200 µg/mL) presented on UV spectrum, after background subtraction (buffer and isomangiferin 100 µg/mL spectrum). Arrows indicate the increase in peaks formed with increasing elastase concentration.

The course of the formation of complexes between isomangiferin and elastase was analyzed in another experiment, in which a constant concentration of isomangiferin (100 µg/mL) and an increasing concentration of the enzyme (2.5–200 µg/mL) were used (Figure 5).

From the resulting absorbance curves, it can be concluded that with increasing enzyme concentrations, a dose-dependent interaction between the enzyme and isomangiferin occurred. The assay revealed two peaks at 286 nm and 374 nm. Additionally, a decrease in absorbance was observed at 318 nm. The peaks at 286 and 374 nm indicated the formation of an isomangiferin–enzyme complex. The most significant interaction was located in the range 374 to 390 nm, but this complex prevents monitoring of product formation change at λ = 410 nm.

In conclusion, the interaction between elastase and isomangiferin occurred; however, calculation of IC_50_ values was impossible with the spectrophotometric method. Therefore, additional studies confirming the antielastase effect of isomangiferin were performed.

**Figure 6:**
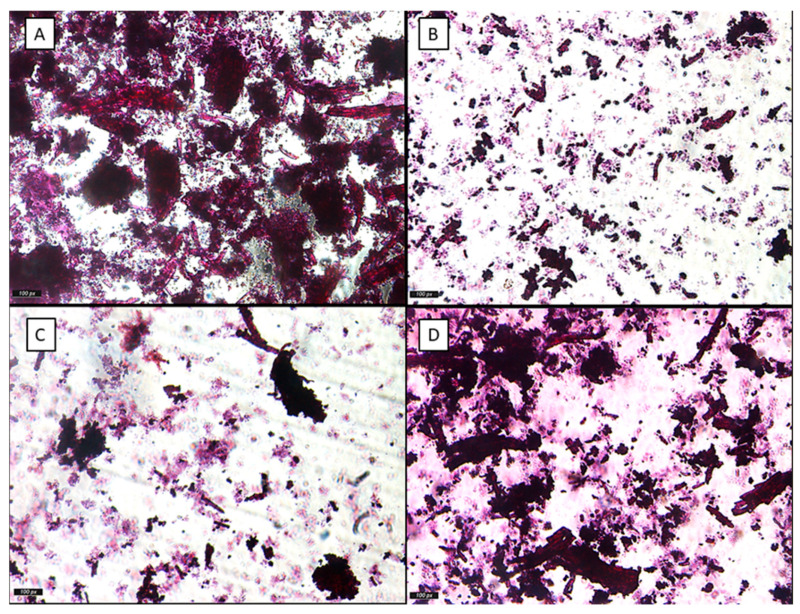
Inhibition of elastase-induced degradation of elastin fibers by isomangiferin. (**A**) elastin + isomangiferin 143 µg/mL; (**B**) elastin + elastase 25 µg/mL; (**C**) elastin + elastase 25 µg/mL + isomangiferin 70 µg/mL; (**D**) elastin + elastase 25 µg/mL + isomangiferin 143 µg/mL. A Leica OMIL LFO FLUO inverted microscope equipped with a Leica OF 310 FX(PC) camera with 100 × magnification was used for observation. Scale: 100 px = 108.52 µm.

In the first experiment, elastin fibers were used. A suspension of the fibers was incubated with elastase in the presence of various isomangiferin concentrations. The results are presented in Figure 6. Elastin fibers are white in color, but in the experiment, they were stained with a selective dye, which allowed observation of both undigested elastin fibers (mostly large dark-purple fragments of fibers) and small digested elements, visible as tiny purple dots. The lighter the color of the fibers, the more elastin fibers were degraded by the enzyme. Figure 6A shows the negative control, where only fibers suspended in the buffer are presented. Figure 6B shows the positive control, where an enzyme was added to the elastin fibers. Figure 6C,D depict elastin fibers incubated in the presence of increasing concentrations of isomangiferin (70 and 143 µg/mL) and elastase (25 µg/mL). The sample treated with 70 µg/mL isomangiferin showed a visually smaller amount of large fiber fragments and an increased number of small digested pink particles compared to the sample with 143 µg/mL isomangiferin.

Image analysis was performed to quantitatively confirm the observations from microscopic images. The positive and negative controls showed the most extreme values of maximal length of the elastin fragments (Table 2). Size distribution analysis of D_10_, D_50_, and D_90_ confirms increasing length and quantity of undigested elastin fragments, especially in D_90_ values.

The positive control has an intermediate mean length (~49 µm) with a relatively narrow D_10_–D_90_ span (≈22–81 µm), consistent with a moderately polydisperse but not extremely broad distribution. Isomangiferin 143 µg/mL shows a slightly higher mean and clearly higher D_90_ than the positive control, indicating a longer tail of large rods; the increased SD supports this broader distribution. Isomangiferin 70 µg/mL is overall finer (lower mean, median, and D_50_) and less coarse at the upper tail than 143 µg/mL, but its D_90_ is still close to the positive control, suggesting some longer rods remain even at the lower concentration. The negative control is markedly coarser: the mean and D_50_ are roughly 2–3× higher than any isomangiferin condition, and D_90_ extends beyond 200 µm, matching a very broad, coarse distribution.

Both isomangiferin concentrations and the negative control differ statistically from the positive control in mean rod length. The smallest *p* value is for the negative control, reflecting the very large separation in mean and distribution (it is clearly the coarsest group). The 70 µg/mL group, despite having a mean somewhat lower than the positive control, is also significantly different statistically, reflecting a shift toward shorter rods and lower D_50_.

The negative control exhibited a very coarse distribution, while the positive control/isomangiferin samples exhibited a finer but still distinct distribution, with the 143 µg/mL group skewing toward longer rods and the 70 µg/mL group toward shorter ones relative to the positive control. The obtained results showed that isomangiferin can inhibit elastase activity. The amount of undigested elastin fragments increased with increasing concentration of isomangiferin.

**Figure 7:**
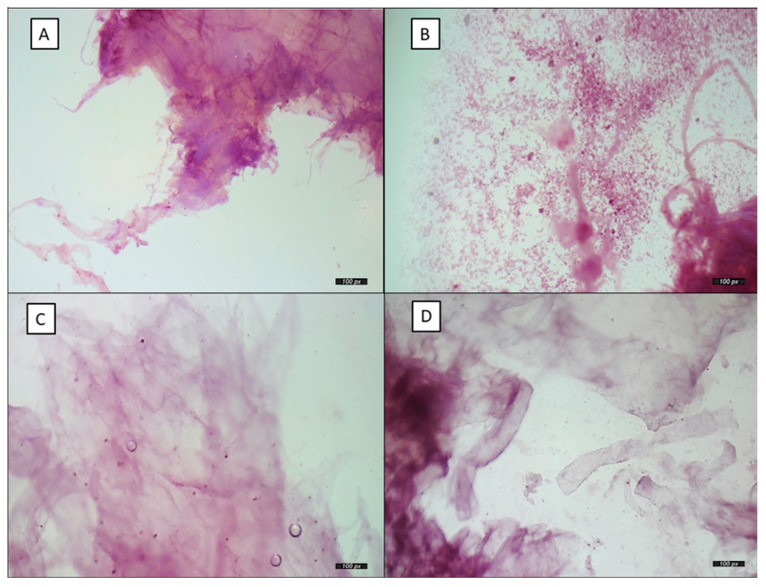
Inhibition of elastase-induced degradation of pork tendons by isomangiferin. (**A**) Pork tendons; (**B**) pork tendons + elastase 50 µg/mL; (**C**) pork tendons + isomangiferin 200 µg/mL; (**D**) pork tendons + elastase 50 µg/mL + isomangiferin 200 µg/mL. An OPTA-TECH binocular with 100 × magnification was used for observation. Scale: 100 px = 108.52 µm.

Elastase inhibition by isomangiferin was also confirmed in another experiment with porcine tendons. The results are presented in Figure 7. Elastin fibers were stained purple. Figure 7A (tendons and buffer), presenting a negative control, shows whole, undigested elastin fibers from tendons. The presence of isomangiferin for 8 h incubation (Figure 7C) did not affect the tendon structure. Figure 7B presents partly digested elastin fibers after incubation with elastase (positive control). Digested fibers are seen as purple spots suspended in the reaction mixture. The enzyme concentration used in this experiment did not destroy all porcine tendons, since not all macromolecules were substrates for elastase. Figure 7D shows undigested fibers and a rare number of purple dots, representing small fragments of digested elastin fibers. This indicates that isomangiferin was capable of significantly inhibiting elastase.

#### 2.3.2. Docking Studies

To elucidate the structural basis of isomangiferin activity and to compare its binding mode with that of mangiferin, molecular docking calculations were performed.

Docking was carried out using the GNINA version 1.3.1 software, which is based on AutoDockVina sampling and, in addition to conventional scoring functions, employs a trained convolutional neural network (CNN) to evaluate and rank binding poses, thereby increasing the likelihood of identifying the most accurate binding mode.

The estimated free energy of interaction was −30.42 kcal/mol for isomangiferin and −28.38 kcal/mol for mangiferin. The CNN score, ranging from 0 to 1 and reflecting the quality of the predicted binding pose, reached 0.61 for isomangiferin and 0.81 for mangiferin. The CNN affinity values, corresponding to the predicted binding affinity expressed as pK, were 3.8 and 4.1 for isomangiferin and mangiferin, respectively.

**Figure 8:**
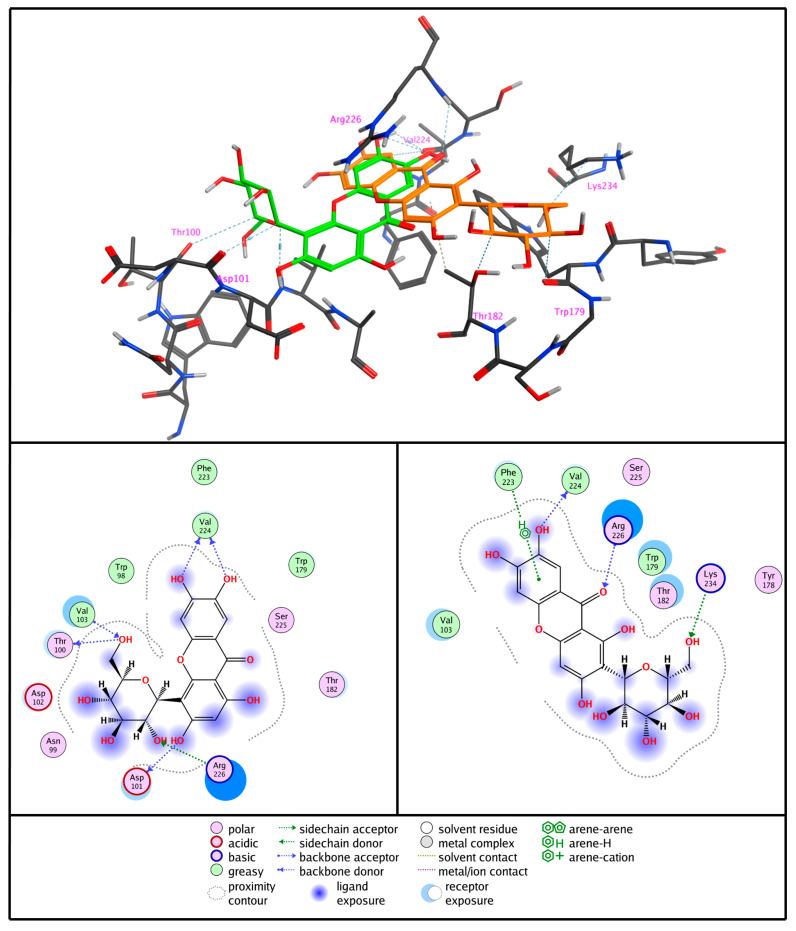
Comparison of the binding modes and protein–ligand interactions of isomangiferin (green stick model and left scheme) and mangiferin (orange stick model and right scheme).

Both compounds bind to porcine pancreatic elastase, not directly within the catalytic site but in the S4 subpocket, according to Bode et al. [27]. In this region, they both form hydrogen bonds with Val224 and Arg226, while the glucopyranosyl moiety of mangiferin is oriented toward the S5–S6 subpocket (Figure 8). Due to its distinct L-shaped structure, isomangiferin adopts a slightly different binding mode from mangiferin. The condensed three-ring xanthone system is rotated by approximately 45° compared to mangiferin, while still maintaining interactions with the backbone of Val224 and Arg226. This orientation directs the sugar moiety in the opposite direction, enabling additional interactions with Thr100 and Asp101 (Figure 8). Owing to the greater number of hydrogen bonds, the interaction caused by isomangiferin is expected to exhibit at least similar stability to that caused by mangiferin. However, additional research on interaction creation is necessary in this case.

### 2.4. Antihyaluronidase Activity

**Figure 9:**
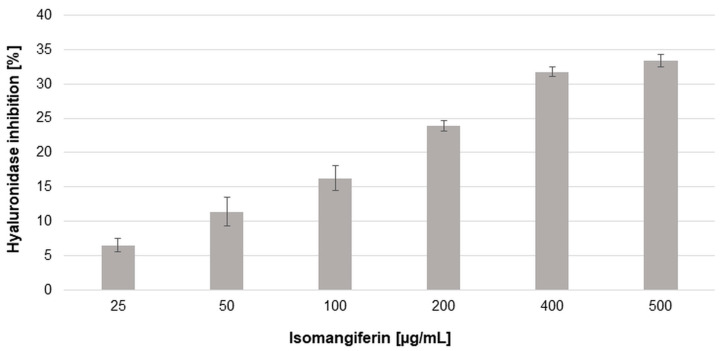
The influence of isomangiferin on the inhibition of hyaluronidase activity. The results are presented as mean values with standard deviations (±SD) obtained in three experiments performed in three replicates (*n* = 9). Error bars indicate standard deviations.

Analysis of the effect of isomangiferin on hyaluronidase activity showed that even at low concentrations of the compound (25 µg/mL), a reduction in enzyme activity was observed (Figure 9). Further increasing the isomangiferin concentration up to 100 µg/mL resulted in a gradual and systematic increase in enzyme inhibition. As the isomangiferin concentration increased, the inhibitory impact started to slow down. At concentrations of 400–500 µg/mL, hyaluronidase activity only decreased by 35%; thus, in this experiment, the exact IC_50_ value (the concentration causing 50% enzyme inhibition) could not be determined. The rapid decline in hyaluronidase activity induced by even low concentrations of isomangiferin suggests that the xanthone interacts with the enzyme in a different place than the active center. While this other binding site can be saturated by the isomangiferin, the catalytic active site can remain functional. Therefore, a detailed analysis of the type of inhibition should be evaluated in further studies. Nevertheless, the obtained data allow for a preliminary assessment that isomangiferin exhibits moderate anti-inflammatory properties with respect to the mechanism of hyaluronidase inhibition. It should be emphasized that, depending on the type of tissue and the type of hyaluronidase, the effect of isomangiferin may vary, thus requiring further in-depth in vitro and in vivo studies. Additionally, hyaluronidase is characterized by high tissue specificity. The presented data were obtained in vitro, and it can be assumed that in vivo studies might yield different results. The inhibitory effect could be more pronounced or observed at different isomangiferin concentrations due to the complex metabolic mechanisms and the presence of other factors regulating inflammatory processes in the body [23].

The obtained results suggest that isomangiferin may have anti-inflammatory potential, as hyaluronidase activity is significantly associated with extracellular matrix degradation and the exacerbation of inflammatory processes in tissues [22,28].

## 3. Discussion

Many polyphenolic compounds in nature are effectively used as supportive or therapeutic agents in diseases related to oxidative stress and inflammation. Natural compounds like curcumin, polyphenols from *Tea sinensis*, quercetin, or resveratrol can modify the expression of genes responsible for inflammation and regulate the immune system response [29,30].

Many studies have been conducted on the biological effects of plant extracts and individual compounds derived from mango and honeybush, including mangiferin and isomangiferin [2,3,4,31]. So far, most of the research refers only to the biological properties of mangiferin. Both compounds are C-glycosides (1,3,6,7-tetrahydroxyxanthone) and differ only in glucose location (Figure 1). They have similar chemical properties, but can differ in pharmacological properties, much like vitexin and isovitexin [32].

In this work, we focused on the biological activity of isomangiferin, in particular its antioxidant and anti-inflammatory effects, especially regarding elastase and hyaluronidase inhibition.

The conducted studies confirmed the high antioxidant activity of isomangiferin. Xanthone’s ability to reduce iron ions and inhibit radicals was higher than that of ascorbic acid. Furthermore, isomangiferin did not exhibit toxic effects on human keratinocytes HaCaT. Isomangiferin demonstrated a significant protective effect on the cells exposed to menadione-induced oxidative stress, preventing cell death. The effect was dependent on isomangiferin concentration and incubation time. The highest concentration of the compound administered for 4 h was effective and protected the cells from menadione-induced cell death (cell survival was approximately 90%).

In this study, isomangiferin interacted with elastase, an enzyme responsible for many biological functions [14,15,16,17,18,19]. However, the overlapping absorbance maxima of the substrate, product, and the resulting isomangiferin-elastase complex made it impossible to measure the IC_50_ parameter using the spectrophotometric method.Thus, alternative methods were used. Two of them were based on the visible degradation of elastin fibers by elastase. The studies using biological material (pork tendons) and laboratory elastin demonstrated the inhibition of elastin fiber degradation by isomangiferin in the presence of elastase. The degree of elastase inhibition depended on the concentration of isomangiferin, which prevented the digestion of elastin in the samples. These methods failed to achieve an isomangiferin concentration that limited elastase activity by 50%; however, they enabled the inhibitory effect of isomangiferin to be assessed visually and through analysis of fiber size images. To assess whether isomangiferin or mangiferin is more potent as an elastase inhibitor, the IC_50_ value should be assessed. HPLC or fluorometric assays may be useful in overcoming the overlapping absorbance spectra problem [33,34].

In this study, we decided to use molecular docking to estimate how the structural arrangement may influence the isomers’ (mangiferin and isomangiferin) affinity to the elastase. Calculations indicated a more favorable binding energy for the complex formed between isomangiferin and porcine elastase compared with mangiferin. In the absence of an experimentally determined IC_50_ value, this suggests that the activity of isomangiferin was at least comparable to that of mangiferin. Relocation of the glucosyl substituent in isomangiferin from the C2 to the C4 position of the xanthone scaffold (vs. mangiferin) not only alters the overall molecular geometry from a more linear to an L-shaped conformation, but also markedly changes the spatial distribution of hydrogen-bond-donating hydroxyl groups. As a consequence, isomangiferin can be able to interact with a larger number of amino acid residues, thereby promoting the formation of a more stable ligand–enzyme interaction. Previous in vitro research conducted on mangiferin inhibition of elastase revealed that the compound presented a non-competitive type of elastase inhibition. The compound was characterized by affinity for both the free enzyme and the enzyme-substrate complex [9]. In this work, the docking studies showed that isomangiferin can also bind to elastase. This may be particularly important in diseases associated with excessive activity of ECM enzymes, such as tendon degradation or regeneration. Tendons are composed of a large number of collagen and elastin fibers, and extracellular matrix (ECM) enzymes are responsible for their remodeling. Inadequate regeneration of tendons is related to oxidative stress mainly induced by highly processed food and/or tobacco smoke; thus, the antiradical and ECM enzymes inhibiting properties of isomangiferin may have a beneficial effect on tendon condition [15,35].

The studies also confirmed that isomangiferin had a moderate impact on hyaluronidase inhibition. The anti-hyaluronidase effect was seen as dose-dependent. Despite the increased inhibition, the IC_50_ level was not reached, suggesting that isomangiferin binds to the enzyme at a location other than the active site and only partially blocks its function. It is worth emphasizing that the hyaluronidase used in the experiment was derived from bovine testicles, and this enzyme is highly tissue-specific; therefore, in other tissues or organs, the inhibitory effect of isomangiferin may be stronger or occur at different concentrations. These results indicate a moderate potential of isomangiferin, but a full understanding of its mechanism of action requires further in-depth studies, both in vitro and in vivo. This mainly concerns the effect of isomangiferin on the ECM enzymes in the context of inhibiting/reducing inflammatory processes.

Isomangiferin is a natural compound with many beneficial properties that can be pharmacologically useful in supporting the treatment of diseases caused by increased activity of enzymes in the extracellular matrix. The obtained results showed that the compound has the potential to protect cells against ROS and elastin damage/degradation. Thus, isomangiferin can potentially be applied in the cosmetics industry as an antiaging ingredient. Additionally, the alternative methods for assessing isomangiferin activity using elastin fibers and analysis of fiber size images may be useful in the preliminary study of a compound’s inhibitory effect on elastase activity, especially when the spectral maxima of the substrate, product, and the formed complex overlap.

However, before isomangiferin can be commercially used, further studies investigating its affinity for macromolecules like enzymes and confirming its safety are necessary.

## 4. Materials and Methods

### 4.1. Materials

Isomangiferin, DMSO, ABTS, DPPH, ascorbic acid, SANA, elastin staining solution for microscopy, TPTZ, oxaliplatin, acetic acid, methanol, pancreatic elastase from pork pancreas, and sodium acetate were bought from Sigma-Aldrich FineChemicals (St. Louis, MO, USA). Ferric chloride × 10 H_2_O was sourced from WARCHEM Sp. z o.o. (Zakręt, Poland). Tris-HCl buffer (pH 8.0; 10 mM) was obtained from A&A Biotechnology (Gdańsk, Poland). Elastin for laboratory use was sourced from MP Biomedicals, LLC (Santa Ana, CA, USA). Menadione, MTT reagent, Dulbecco’s Modified Eagle’s Medium (DMEM), fetal bovine serum (FBS), and penicillin and streptomycin were sourced from Merck Millipore (Burlington, MA, USA). Human keratinocytes (HaCaT) were obtained from the American Type Culture Collection (ATCC, Manassas, VA, USA).

The biological material used was a pork shoulder purchased commercially from Lewiatan (Gdańsk, Poland).

### 4.2. Methods

#### 4.2.1. Antioxidant Assays

The analyses were carried out using colorimetric methods with a 96-well plate and a spectrophotometer (Epoch Biotek, Santa Clara, CA, USA). The results of antioxidant and reduction power expressed as IC_50_ values of isomangiferin and ascorbic acid were calculated with GraphPad Prism v. 9.0.0. From each sample, a blank sample was subtracted, which contained a buffer and the appropriate concentration of the compound: isomangiferin or ascorbic acid. For the ABTS and DPPH, ascorbic acid was used as a standard. In the FRAP assay, a calibration curve was prepared with the use of ascorbic acid. For the antioxidant assays, isomangiferin and ascorbic acid were freshly diluted in 25% DMSO (*v*/*v*); the control sample was composed of DPPH/ABTS solution and 25% DMSO (*v*/*v*).

##### FRAP Assay

The antioxidant activity of isomangiferin was determined using the spectrophotometric method of iron ions reducing power (FRAP) [36]. The principle of the method is to determine the ability to reduce Fe^3+^ ions to Fe^2+^ ions, which are complexed by TPTZ. The resulting product gives an intense blue color with an absorbance maximum at 593 nm. The FRAP reagent was prepared freshly just before use (0.3 M acetate buffer: 10 mM TPTZ in 40 mM HCl:20 mM FeCl_3_·6H_2_O in a ratio of 10:1:1). A series of isomangiferin dilutions (0.5–400 μg/mL) were mixed with FRAP solution (30 and 170 μL, respectively) and incubated for 15 min at room temperature. The control was composed of FRAP reagent and 25% DMSO (*v*/*v*). The percentage of reduced iron ions was read from the calibration curve plotted for the standard ascorbic acid (1–1000 µg/mL).

##### ABTS Assay

The radical scavenging ability was assessed with the ABTS test [37]. The control was composed of ABTS and 25% DMSO (*v*/*v*). In total, 30 μL of different concentrations of isomangiferin (0.5–400 μg/mL) was mixed with 170 μL of ABTS solution (2 mM ABTS diammonium salt, 3.5 mM potassium persulfate) and 50 μL of water. After 20 min of incubation at 30 °C in the dark, the absorbance was observed at λ = 750 nm. ABTS inhibition was calculated according to the following equation:


ABTS inhibition (%) = [(Acontrol − Asample)/Acontrol] × 100%

##### DPPH Assay

The DPPH assay was performed with ascorbic acid as a standard [37]: 50 μL of different concentrations of isomangiferin (0.5–400 μg/mL) was mixed with 50 μL of 0.06 mM DPPH methanol solution. The control sample was composed of DPPH solution and 25% DMSO (*v*/*v*). The reaction mixtures were incubated in the dark at room temperature for 30 min. The absorbance was observed at λ = 517 nm and calculated according to the following equation:


DPPH Inhibition (%) = [(Acontrol − Asample)/Acontrol] × 100%

#### 4.2.2. Cell Culture Assay

The human keratinocytes (HaCaT) were seeded in 96-well plates (5 × 10^3^ cells/well) and incubated for 24 h in a humidified 5% CO_2_ incubator (37 °C) in DMEM supplemented with 10% (*v*/*v*) FBS, 100 units/mL of penicillin, and 100 μg/mL of streptomycin.

The MTT assay was used to analyze the cytotoxic effect of isomangiferin itself and to assess the protective effect of isomangiferin from oxidative stress in the test with menadione, expressed as cell viability (%). Oxaliplatin was used as a positive control (at concentrations of 10–150 μg/mL).

Briefly, the cells were seeded and incubated for 24 h. The isomangiferin dilutions were added to the cells (40–200 μg/mL). The concentration of DMSO in the control sample (solvent of the isomangiferin) was 0.5% (*v/v*). After 24 h, the MTT reagent was added to the plates. The formazan crystals were dissolved in isopropanol, and the absorbance was measured at 570 and 650 nm.

In the next test assessing the protective effect of isomangiferin on HaCaT cells exposed to oxidative stress, menadione was used. During this experiment, three concentrations of isomangiferin, 40, 80, and 100 μg/mL, were used, which were incubated with the cells for 2 or 4 h. After this time, menadione at a concentration of 7 μg/mL was added and left for further 24 h incubation. An MTT assay was then performed to determine the percentage of cells that survived compared to the control.

Three experiments in at least three repetitions were performed. The data are expressed as the percentage of cell viability calculated in GraFit 7.0 software (Erithacus Software, West Sussex, UK).

#### 4.2.3. Antielastase Assay

##### Kinetic Assay

The elastase inhibition property of isomangiferin (in 25% DMSO (*v*/*v*)) was assessed spectrophotometrically according to the method of Thring et al. [26], modified by Ochocka et al. [4]. SANA was used as a substrate. The reaction mixture was composed of Tris-HCl buffer (pH 8.0), 25 µg/mL porcine pancreatic elastase, and different concentrations of isomangiferin (0–500 µg/mL). The reaction mixtures were incubated with the compounds for 15 min at room temperature in 96-well plates (Epoch, BioTek System, Santa Clara, CA, USA). The addition of the substrate started the reaction. The changes in the product formation were recorded spectrophotometrically at λ = 410 nm every 20 s for 20 min (Epoch, BioTek Instruments, Santa Clara, CA, USA). The results were analyzed in the GraphPad Prism v. 9.0.0. (GraphPad Software, San Diego, CA, USA) and Microsoft Excel. The control was composed of Tris-HCl buffer (pH 8.0) and SANA. Oleanolic acid was used as a standard.

##### Interaction Elastase—Isomangiferin Tests

Since it was impossible to determine the degree of inhibition caused by isomangiferin on elastase using the spectrophotometric method described by Thring et al. [26], an additional study was initiated to determine which compounds participating in the enzymatic reaction interact with isomangiferin. Therefore, a reaction mixture was prepared in a specific combination. Each mixture was analyzed by the spectrophotometric method, assessing the spectrum and determining the absorbance maxima.

The total volume of the test samples was 200 µL. The composition of the individual reaction mixtures was as follows:

- SANA [1.0 mg/mL] + buffer;
- Isomangiferin [100 µg/mL] + buffer;
- Elastase [25 µg/mL] + buffer;
- Isomangiferin [100 µg/mL] + SANA [1.0 mg/mL] + buffer;
- Isomangiferin [100 µg/mL] + elastase [25 µg/mL] + buffer.

The results were analyzed after subtracting the buffer spectrum.

Since it was shown that SANA did not interact with isomangiferin, complex formation of isomangiferin with elastase was analyzed. The constant concentration of isomangiferin [100 µg/mL] was incubated with elastase [2.5–200 µg/mL].

The composition of the individual reaction mixtures was as follows:

- Isomangiferin [100 µg/mL] + buffer;
- Isomangiferin [100 µg/mL] + elastase [2.5–200 µg/mL] + buffer.

The results were analyzed after subtracting the buffer + isomangiferin [100 µg/mL] spectrum.

##### Elastin Fiber Assay

Commercial elastin was used in this study to obtain reproducible results. This analysis aimed to prove that isomangiferin has an inhibitory effect on elastase activity.

Tris-HCl buffer (0.2 M, pH 8.0), isomangiferin (concentration range: 70–143 µg/mL), and enzyme (25 µg/mL) were added to elastin fibers (5 mg/mL). Incubation was carried out for 60 min at 100 rpm at 37 °C using a shaker incubator. After one hour, an elastin staining solution was added to the samples for 30 min. The samples were observed under 100 × magnification using a Leica OMIL LFO FLUO inverted microscope equipped with a Leica OF 310 FX(PC) camera (Leica, Wetzlar, Germany). Negative control was composed of Tris-HCl buffer, isomangiferin, and elastin fibers. Positive control was composed of Tris-HCl buffer, elastase, and elastin fibers.

Image analysis was conducted for the following samples: positive control, negative control, and isomangiferin at concentrations of 143 µg/mL and 70 µg/mL. Particle size characterization was performed in accordance with the European Pharmacopoeia (Ph. Eur.) method 2.9.37 (Optical Microscopy) and ISO 9276 guidelines. Due to the presence of particle agglomeration and insufficient image contrast, automated image analysis was not feasible. Therefore, particle length measurements were performed manually. Individual particle dimensions were determined by manually tracing line segments corresponding to particle length directly on the acquired images. These measurements were subsequently quantified using the image analysis software Gwyddion (version 2.71). The resulting dataset was processed using Microsoft Excel. Descriptive statistics, including mean, median, minimum, and maximum particle lengths, were calculated. Additionally, particle size distribution parameters D_10_, D_50_, and D_90_ were determined. To assess method reproducibility, particle length data within each sample group were statistically compared. Welch’s two-sample *t*-test (two-sided, assuming unequal variances) was applied to evaluate differences between concentrations and control samples. For all comparisons, *p* > 0.7 at a significance level of α = 0.05, indicating no statistically significant differences between datasets, which confirms the measurement protocol reproducibility. Furthermore, the datasets for each type of sample were compared with Welch’s two-sample *t*-test using a positive control as a reference sample.

##### Tendons Assay

The tendons visible in the pork shoulder fragment were dissected with a scalpel and separated from the skeletal muscles. To increase the surface area of contact between the enzyme and the biological material, the obtained tendons were ground using a mortar and pestle. Immediately after preparation, the obtained biological material was placed in a twelve-well plate and suspended in Tris-HCl buffer (0.2 M, pH 8.0). Isomangiferin (200 µg/mL) and elastase (50 µg/mL) were added to the samples, which were incubated in a shaker incubator at 100 rpm for 8 h at 37 °C. Next, elastin dye was added and left for 30 min. After this time, samples were observed under 100 × magnification using an OPTA-TECH binocular. The negative control consisted of tendons and buffer, and the positive control consisted of tendons, buffer, and elastase (50 µg/mL).

The tendon assay was performed only to illustrate the possible antielastase properties of isomangiferin.

##### Docking Study Assay

As target proteins, X-ray structures of porcine pancreatic elastase (PDB code 1ELE) obtained from the Protein Data Bank were selected among other elastase models based on cross-validation results according to the presented methodology.

The protein structure was prepared with Molecular Operating Environment (MOE) 2022.02 software using the “QuickPrep” tool, which includes the correction of structural errors, protonation, and calculation of partial charges, completed with energy minimization in the Amber: EHT force field. For the docking calculation, all water molecules were deleted. Ligand structures were downloaded from the PubChem (https://pubchem.ncbi.nlm.nih.gov/, accessed 29 March 2026) database (mangiferin: CID 5281647, isomangiferin: CID 5318597) and optimized in the Amber: EHT force field using MOE. After preparation, protein and ligand structures were saved in mol2 file format to preserve calculated charges.

Docking was performed using GNINA v1.3.1 [38]. Box size was center x = 36.15, y = 19.85, z = 38.15, and dimensions x = 41, y = 31, z = 31 A. Exhaustiveness was set to 64. The Autodock4 scoring function was utilized as it has been found to give better cross-validation results than the standard vina scoring function. Convolutional neural net (CNN) scoring option was left at the default rescore. The calculations were performed four times, and for further analysis, the pose with the highest CNN_scoring (value closest to 1) was selected. The analysis and visualization of the obtained results were performed with MOE software.

#### 4.2.4. Antihyaluronidase Assay

The hyaluronidase inhibitory activity of isomangiferin was investigated using the method previously described by Kaessler et al. [39] with slight modifications [4]. The reaction mixture was incubated for 10 min in a water bath with different concentrations of isomangiferin. Afterwards, to start the reaction, hyaluronic acid (phosphate buffer 300 mM, pH 5.35) was added. The undigested substrate was precipitated with bovine acid albumin, and the measurement was performed at λ = 600 nm. Oleanolic acid was used as a standard.


Hyaluronidase activity = A_600 nm (hyaluronic acid + hyaluronidase)/A_600 nm (hyaluronic acid)

### 4.3. Statistical Analysis

Statistical analysis was performed with the GraFit 7.0 software (Erithacus Software, West Sussex, UK). All data are expressed as mean values ± standard deviation (±SD). Student’s *t*-test with Welch’s modification (Welch’s *t*-test) was used to compare the results with the control samples. Data normality and homogeneity of variances were evaluated with Shapiro–Wilk and Levene’s tests, respectively. The statistical significance was set at *p* < 0.05.

## 5. Conclusions

The results obtained in this work indicate that isomangiferin is a reactive molecule. Preliminary research indicated that isomangiferin has the potential to limit enzyme activity, specifically elastase and hyaluronidase (in a moderate manner). These enzymes are responsible for the degradation of macromolecules present in the extracellular matrix and inflammatory processes. Additionally, isomangiferin is able to scavenge free radicals and protect cells from ROS. Further in-depth studies on enzyme inhibition by isomangiferin and other biological actions of this compound are necessary both in vitro and in vivo, including its protective effect on macromolecules.

## Figures and Tables

**Table 1:** Antioxidant properties of isomangiferin and ascorbic acid expressed as IC_50_ value [µM].

Assay	Isomangiferin	Ascorbic Acid
FRAP	70.44 ± 1.4 *	315.85 ± 4.49
DPPH	9.57 ± 0.05 *	35.83 ± 0.45
ABTS	27.04 ± 1.8 *	164.6 ± 3.29

Ascorbic acid was used as the standard substance. The results are presented as the mean values with standard deviations (±SDs) calculated from three independent experiments across three repetitions (*n* = 9). Significant differences relative to standard ascorbic acid are marked with an asterisk “*” (Welch’s *t*-test, *p* < 0.05).

**Table 2:** Length [μm] of analyzed particles: elastin fibers after incubation with elastase and isomangiferin.

Sample	*n*	Mean	Mediana	Minimal	Maximal	STD	*p* Value	D_10_	D_50_	D_90_
Positive control	178	48.8	40.1	15.7	187.8	31.1	-	22.4	40.1	81.2
Isomangiferin 143 µg/mL	288	56.8	41.7	9.7	373.0	53.4	<0.004 *	18.3	41.7	111.7
Isomangiferin 70 µg/mL	183	39.5	25.8	8.8	235.3	35.8	<0.00002 *	14.8	25.8	80.1
Negative control	118	113.7	96.3	12.7	477.1	83.1	<0.000001 *	31.7	96.3	208.5

Negative control: elastin + isomangiferin 143 µg/mL; Positive control: elastin + elastase 25 µg/mL; Isomangiferin 70 µg/mL: elastin + elastase 25 µg/mL + isomangiferin 70 µg/mL; Isomangiferin 143 µg/mL: elastin + elastase 25 µg/mL + isomangiferin 143 µg/mL; * denotes statistically significant difference in comparison to a positive control (Welch’s *t*-test).

## Data Availability

All data are included in the manuscript.
